# Optimising remanufacturing decision-making using the bees algorithm in product digital twins

**DOI:** 10.1038/s41598-023-27631-2

**Published:** 2023-01-13

**Authors:** Mairi Kerin, Natalia Hartono, D. T. Pham

**Affiliations:** 1 Department of Mechanical Engineering, School of Engineering, College of Engineering and Physical Sciences, University of Birmingham Edgbaston Campus, Birmingham, UK; 2 Department of Industrial Engineering, University of Pelita Harapan, M.H. Thamrin Boulevard 1100 Lippo Village, Tangerang, 15811 Indonesia

**Keywords:** Mechanical engineering, Environmental impact, Computational science

## Abstract

Remanufacturing is widely recognised as a key contributor to the circular economy (CE) as it extends the in-use life of products, but its synergy with Industry 4.0 (I4.0) has received little attention when compared to manufacturing. An agglomeration of I4.0 technologies and methodologies is reflected in the emerging digital twin (DT) concept, which has been identified as a life-extending enabler. This article captures the design and demonstration of a DT model that optimises remanufacturing planning using data from different instances in a product’s life cycle. The model uses a neural network for remaining useful life predictions and the Bees Algorithm for decision making within a DT. The model is validated using a real case study. The findings support the idea that intelligent tools within a DT can enhance decision-making if they have visibility and access to the product’s current status and reliable remanufacturing process information.

## Introduction

Remanufacturing, as defined by the British Standards Institution^1^, is a key strategy to increase resource circularity and sustainability^2^. Research into smart remanufacturing systems is increasing in popularity, with recent discussions focusing on how technology from Industry 4.0 (I4.0), as applied in the manufacturing sector, can increase efficiency and digitalisation in remanufacturing^3–5^. However, differences between the two sectors means that direct application of I4.0 technology from the former to the latter may not be practicable^6^.

‘Core’ is used to describe a used part or product, that can have a second life after being re-processed. This is a generic term that crosses industries and sectors. The supply of core, which is the input to the remanufacturing process, is unpredictable in terms of quantity and quality. Some remanufacturers actively manage the process of core acquisition, while others do not have that luxury^7^. Quality ‘classes’ are regularly used to categorise and incentivise return, but classification is often performed at the core supplier or collection sites^7^ with the business having already invested in what may end up being a non-remanufacturable product. Cores may be dirty, distorted or worn. The original product may not have been designed with disassembly in mind or may have been built with easy to assemble, but difficult to disassemble, one-way or irreversible fittings. Additionally, depending on customer demand and remanufacturing business capabilities, partial, targeted and destructive disassembly operations can complicate routings further^8^.

The remanufacturing process steps that follow disassembly often vary and can lead to hybrid assemblies combining new and refurbished components. The demand for remanufactured products and components is also highly volatile^9^. Variability in supply and demand makes for complex business models and processes that require flexibility that until recently could only be realised manually. This has led to a sector that exhibits low levels of technology utilisation^10^. However, as technology and information systems advance and become more accessible, new opportunities arise to improve the management of uncertainties by providing near real-time information about a product’s in-use performance, making predictions around its EoL time and state, and autonomously assessing the multidimensional needs of the core and the business^11^.

Following a literature review in section “Literature review”, an automated Decision-Making Module for remanufacturing is developed, discussed and evaluated using a case study. Section “Automated decision-making module model” reports on the sustainable performance metrics that could be used to make an informed decision in remanufacturing. Section “Case study” covers the decision-making module model and its structure. Sections “Results” and “Discussion”, respectively, present and discuss the results of using the DMM in a case study. Section “Conclusion” summarises the paper and outlines further research opportunities.

## Literature review

Only 6% of the work reviewed by Rizova et al.^12^ has attempted to accommodate more than two remanufacturing uncertainties in one decision-making process. Using the list of uncertainties by Rizova et al.^12^, namely, (1) demand for remanufactured products, (2) returns quality, (3) returns quantity, (4) lead time, (5) returns timing and (6) routing, it has already been shown that product DTs, which are digital models with details of how the product was used throughout its service life, can offer information on 2, 3, 4 and 5. On the other hand, a process DT, which models the remanufacturing operation, is likely to yield data enabling remanufacturers to handle 6^11^. In combining the two DTs to create an end-to-end DT, all uncertainties could be contested to some degree. This highlights how significant the transformation of remanufacturing decision-making could be following the integration of DTs and the impact they could have on certainty and stability in this sector.

Ultimately, the output of the decision-making activity required by remanufacturers, as a function of the DT, is twofold. First, a decision should be made on whether a product should be recovered based on the opportunities available. Second, a set of operational principles is used to optimise the downstream activities for given business objectives. In remanufacturing, these objectives are normally economically based, but environmental targets are becoming more prevalent^13^, and social elements are also emerging^14^.

Each business is likely to have its own set of objectives, but if remanufacturing itself is to be recognised as developing sustainably, it too needs balanced economic, social and environmental performance^15^. This is referred to as balancing the triple bottom line^16^ and further challenges decision-makers, as social and environmental metrics are not always quantitative. To analyse the cost–benefit, the remanufacturing process needs to be fully understood, specifically the disassembly process.

Finding the optimised disassembly routing means solving a non-deterministic polynomial-time complete problem^17^. This type of problem crosses disciplines and appears to be well researched^18^, including in less studied sectors such as remanufacturing, as documented recently in Zhou et al.^19^. There are many manual and automated methods available that can be enhanced or hybridised to improve solution speed, search space and/or accuracy. Nature-inspired algorithms (NA) appear to be the most prominent and include ant, bee, and fruit-fly colony optimisation as well as immune response and particle swam methods.

Metaheuristic algorithms are widely used due to their adaptability and have been shown to be effective when applied to product disassembly models^20,21^. Previous research into resolving disassembly problems using the Bees Algorithm (BA) reports that it is superior in terms of time and quality when seeking to find the optimal solution^20–24^. As a result, the BA has been selected as the tool to extract the disassembly sequence from the information contained in the DT. The BA is a population-based metaheuristic that was inspired by the activities of honeybees when foraging for nectar. The algorithm introduced by Pham et al.^25^ has gained attention due to its successful application across an array of case studies and its use in solving complex optimisation problems faster than the exact method^21,26^.

**Figure 1:**
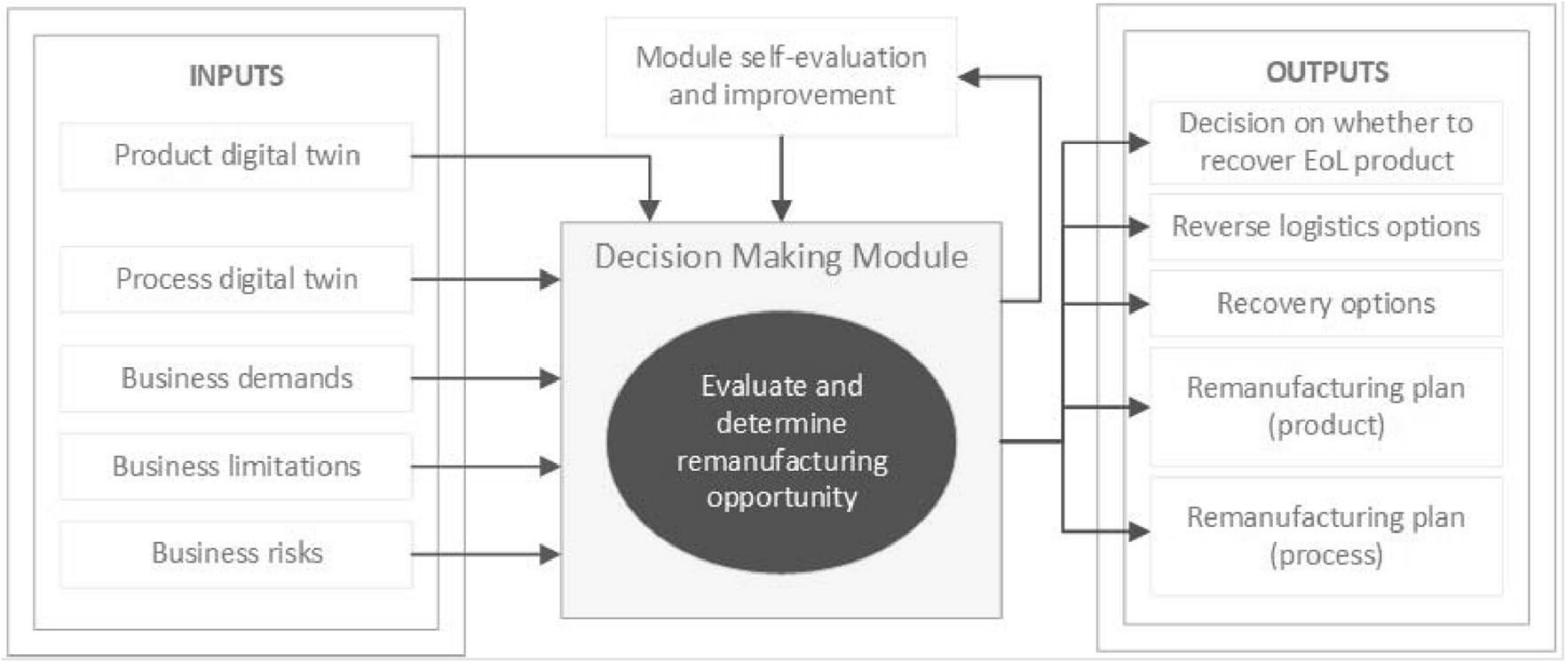
Inputs to and outputs from the decision-making module.

This research explores the inner workings of the decision-making module (DMM) identified in^11^ and depicted in Fig. 1 as a key element of a remanufacturing-centric DT. The questions that will drive this exploration include ‘what are the key variables that need to be incorporated into the DMM to come to a decision?’ and ‘if the remanufacturing business is to be managed to CE principles, how do the outputs of the DT feed into to triple-bottom line assessment and the decision as to whether or not an EoL product is recovered’? A multilevel digital twin (DT), incorporating a product and process DT, has been proposed to improve and automate decision-making for remanufacturing with the aim of improving the visibility of inbound core quantity, quality, demand, and processing opportunities^11^. In this research, a simulated product is digitally connected to a virtual version of the same product to trial the prediction modelling proposed.

## Automated decision-making module model

An end-to-end DT, comprising both product and remanufacturing process DTs, may enable autonomous decision-making. In the DT the decision-making takes place in the DMM.

With reference to (Fig. 1), the DMM will need to accommodate changing business inputs (demands, limitations, and risks) and utilise the beginning-of-life (BoL) and middle-of-life (MoL) data available from the DTs. The outputs are similar to those described in smart recovery decision-making (SRDM)^27^ and include recovery alternatives as well as product and process operational plans. Reverse logistics options are also considered in the module making use of the product and processing locations, an output that was out of scope in Meng et al.^27^. The “optimal” solution depends on the inputs and the applied evaluation criteria.

### Decision-making module inputs and outputs

#### Product DT

A product DT comprises a real product in real space, a virtual product in virtual space, and the connections of data and information that tie the ‘products’ together^28^. The system design used in this work has been described in detail in^11^.

#### Process DT

This couples the production system with its digital equivalent^29^. It is similar to the product DT, as it enables assessment and simulation of potential scenarios feeding key information into the DMM.

#### Business demands, limitations, and risks

Relate to the business expectations and experiences that are not captured or managed by the product or process DTs but will influence the decision.

#### Decision on whether to recover an EoL product

This output indicates to the business the worthiness of product recovery.

#### Reverse logistics options

If the decision to recover an entity or not lies with the business, it can be data-driven. Similarly, if an entity recovery is a foregone activity (contractual, etc.), options on when and where it should be recovered from can be evaluated using DT information. The cost of the reverse logistics operation from an economic, environmental, and social perspective can be assessed using the entity GPS and remanufacturing facility locations.

#### Recovery options

Relates to the choices that the remanufacturer can make to optimise bottom-line benefits once the entity has been made available. Selecting the most appropriate level of disassembly (complete, partial, targeted, destructive) and reassembly (complete, partial, hybrid, built-to-order, build-to-stock) is key to managing a sustainable business.

#### Remanufacturing plan (product)

Relates to the requirements of the existing product to start its second life via the remanufacturing process. The remanufacturing bill of materials (rBoM) can be estimated based on the MoL BoM and quality and performance degradation data from the DT. The remanufacturing bill of process (rBoP), much like the manufacturing BoP, defines the activities that need to happen to translate the product from one state to another, balancing triple-bottom-line performance and technical competence. In this case, the rBoP takes the product from EoL back to BoL. The remanufacturing routings can be generated and may include disassembly and rebuilding sequence plans, process steps, machine operations, materials, slave parts and performance targets. Accurate estimations of remanufacturing bills can benefit line balancing, scheduling and production planning^30^.

#### Remanufacturing plan (process)

This output considers line balancing, scheduling, planning, demand management, core acquisition and inventory holding, relying heavily on the process DT, targeting the best scheme for managing tasks and demands while being limited by technical constraints.

#### Module self-evaluation and improvement

A smart decision-making module can make assessments and carry out improvements to optimise itself, but the system should allow for human intervention and preference selection. In the remanufacturing sector, process planning is heavily dependent on the skills and knowledge of experienced individuals, and this needs to be accommodated^31^.

Before generating the outputs, the decision of whether to remanufacture needs to be made. This will be done by evaluating the triple bottom line as described in the next section.

### Model structure

**Figure 2:**
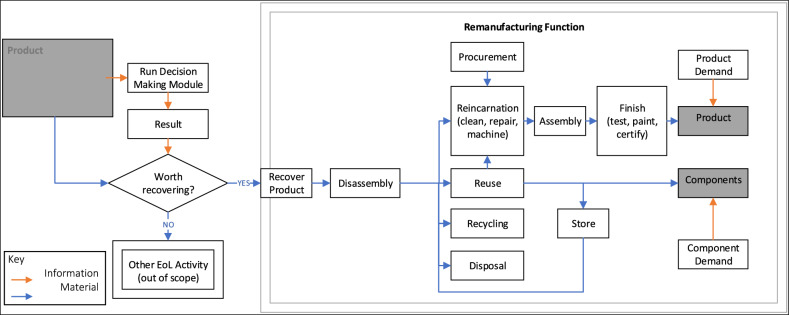
Relationships between DMM and the remanufacturing functions.

The model (Fig. 2) is built on a scenario where a remanufacturing business can choose whether to recover a HiVE from its MoL/EoL user. If the costs associated with recovery and remanufacturing out-weight the business opportunities from a triple-bottom-line perspective, the business will not recover it (other CE practices may be applicable, but these are out of scope). To make this evaluation, the business needs to identify the best remanufacturing strategy that balances profits, environmental effects, and social impact for the given HiVE location and quality from the DT, within the constraints of existing technological capabilities and management policies. Time will be used as a leading variable that will influence economic, environmental, and social measures. It has been assumed that if a process takes longer, it will require more investment and a larger workforce while generating a greater environmental load. Only the benefits to the business from the point that the HiVE becomes available for remanufacturing until it is ready for its second life meeting the expectation that it will be equivalent to, or better than, the manufactured equivalent, not considering multiple life cycles, are in scope.

The model assumes that product recovery will not be triggered without product demand, as the core function of this sector is to remanufacture. The decision of whether to recover the product is evaluated in the DMM. Those that are recovered are first disassembled. Disassembly can be either complete, partial, or targeted with components progressing to a reincarnation phase (normally referred to as remanufacturing but not used in this instance to avoid confusion) that can include any combination of cleaning, repairing, machining, assembly, testing and finishing or filtered into reuse, recycling, disposal, or storage material flows. Reincarnation has been used to describe the processes normally associated with remanufacturing, as BoL assets can be made from EoL products and components.

The demand for products and/or components drives the reincarnation and reuse functions. The entire incarnation of product *i* may be processed through remanufacturing. However, any component *j* of product *i* that needs to be replaced due to damage, missing parts, or needing an upgrade can be sourced from the store or component reuse. Alternatively, new components can be procured (or manufactured). Components with poor quality identified after disassembly may be recycled or disposed of and are lost to the system in this scenario. The routing for each approach is different.

Modifying the formulations of^32^ and Meng et al.^33^ to evaluate the options available utilising real data from the DT prior to product recovery, the economic, environmental and social impacts of remanufacturing the HiVE can be defined. All variables used can be found in the nomenclature. As the economic indicators of remanufacturing are more mature, development will commence.

### Evaluating the economic growth impact in the DMM

In a simplified form from an economic perspective, the potential profit, *P*, is the sales price of the remanufactured product, *SP*, minus the total cost of performing the remanufacturing function, *CRF* (Eq. 1).

1 $$P = SP - CRF$$

With reference to Fig. 2, *CRF* includes the cost incurred by product recovery and disassembly *CPR* and *CPD,* respectively, as well as component storage, *CCS*, reincarnation activities, *CCR*, reuse, *CCU*, recycling *CCC*, and disposal *CCD* (Eq. 2). *CPA* and *CPF* are the cost of product assembly and finishing (including testing, certifying, and final assembly, labelling and painting, etc. assumed to be a single value specific to the product family). Other miscellaneous costs will be assumed to be absorbed by others in this model.

2 $$CRF = CPR + CPD + CCS + CCR + CCU + CCC + CCD + CPA + CPF$$

*CPR* (Eq. 3) can be calculated from the shipping costs, *cs*, of product *i* per kilometre, and the distance, *l* between the product location, available from the DT, and the remanufacturing facility, *f*.

3 $$CPR = cs_{i} l_{f}$$

*CPD* (Eq. 4) relates to whether component *j* needs to be disassembled from the product *nd*, the cost of disassembly per unit of time *cd*, the time it takes to disassemble *td*, a time function *TQ*_1_ given product quality, from the DT, of *q*_*i*_, and the time function *TS* assuming that more time will be taken to disassemble products into components destined for a second life by remanufacturing or reuse *sl*, over those that will be recycled or disposed of, *nsl*. With the CAD, BoM and component relationship information available in the DT, the disassembly sequence can be extracted to match the requirements of the remanufacturing demand. Comprehensive lists of attributes for disassembly in remanufacturing can enable the integration of such systems^34^.

4 $$CPD = \mathop \sum \limits_{j} (nd_{j} cd_{j} td_{j} (TSsl_{j} + nsl_{j} )TQ_{1} )$$

*CCS* (Eq. 5) results from the need to hold components *nh*, which have been identified as having second life potential but cannot be used in the existing product and are also not recycled or disposed of. If component $j$ needs to be stored *nh*, *ch* is the cost of holding per unit of time and *th* is the time in storage, then

5 $$CCS = \mathop \sum \limits_{j} (nh_{j} (ch_{j} th_{j} + cph_{j} ))$$

Following disassembly or recovery from storage, the components can take one of four remanufacturing option routes (decision variables), namely, reincarnation, reuse, recycling, or disposal. *ro*_*x*_ represents the routes where $x \in \left\{ {1,\;2,\;3,\;4} \right\}$ respectively. As described previously, the reincarnation processes, CCR (Eq. 6), in the remanufacturing function refer to the repair, upgrade, rebuild, etc. of the product or hybrid of components to match or better the quality of a new equivalent. Having already been disassembled, the components may need to be cleaned, machined (additive or subtractive) or repaired *nr*. An estimation of the work that may be required could be made in the process DT using the information from the product DT comparing the “as manufactured” with the “current state” instances. Herein offers an opportunity to map product requirements to process capabilities to create suitable production plans. A production line enabled with physical and virtual reconfigurability for individualised product manufacturing, as discussed by Leng et al.^35^, could complement this.

The cost per unit of time in reincarnation is cr. The time tr to complete these reincarnation activities is affected by the quality of the received component. An estimate of product quality is provided by the DT as q_i_*,* but the component quality will depend on the MoL environment and utilisation. A DT at the component level or inferred quality from the performance metrics and/or failure mode data would need to be available to estimate component RUL and quality. This was not demonstrated in the previous chapters, but it is assumed to be possible with improvements in data analytics, diagnostics, and prognostics. To continue, $q_{j} = q_{i}$ in this model and a time function TQ_2_ that translates the impact of the different quality levels on time taken to reach *q*_*min*_ will be applied, where *q*_*min*_ is the minimum quality needed for the component to be successfully incorporated into a remanufactured product. Once ready for reassembly, components can be built into products alongside new (procurement) or spare parts (reuse) if needed. *CCP* (Eq. 7) is the total component purchasing costs where the costs to procure a part or service *cp* to replace one that will be retained for reuse, has failed, is damaged, is consumable, or upgradable (with the original destined for recycling or disposal) or is missing when the product was recovered are also included.

6 $$CCR = ro_{j1} = \mathop \sum \limits_{j} nr_{j} \left( {TQ_{2} cr_{j} tr_{j} } \right) + CCP$$

where

7 $$CCP = \mathop \sum \limits_{j} cp_{j} (ro_{j2} + ro_{j3} + ro_{j4} + m_{j} )$$

where *m* is a missing part.

With the parts already disassembled, there may be cost associated with preparing a component for reuse *CCU* (Eq. 8), related to time *tu* and quality *q*_j_ to feed into either the reincarnation fr_j_ or reuse *fu*_j_ flow. Additionally, as *P* in Eq. (1) only considers the remanufactured product, *CCU* includes the revenue *cru*_*j*_ generated directly from the sale of components.

8 $$CCU = ro_{j2} = \mathop \sum \limits_{j} nu_{j} \left( {TQ_{3} cu_{j} tu_{j} \left( {fr_{j} + fu_{j} } \right) - cru_{j} } \right)$$

The cost of recycling *CCC* (Eq. 9) comes from the sum of the costs minus the material sales revenue *mrc* from the component in question *nc*. Material-level data and component weights from the DT BoM enable this assessment. Product-level recycling is not considered, as it is assumed that the product is only recovered from the end user if it has remanufacturing potential. It is also assumed that this activity is a transaction, and the process of recycling is out of scope.

9 $$CCC = ro_{j3} = \mathop \sum \limits_{j} nc_{j} (cc_{j} - mrc_{j} )$$

Similarly, the cost of component disassembly *CCD* (Eq. 10) includes disposal cost *cdp* for components set for this route only *ndp*. No revenue opportunities are expected from the disposal option. Component weights from the DT may support this.

10 $$CCD = ro_{j4} = \mathop \sum \limits_{j} ndp_{j} cdp_{j}$$

As the aim is to build a unit for the market, the cost of product assembly *CPA* (Eq. 11) with *na* representing the components required to assemble the new product, the total costs per unit of time incurred from assembling is expressed in *ca* and the time is *ta*.

11 $$CPA = \mathop \sum \limits_{j} na_{j} ca_{j} ta_{j}$$

Constraints are similar to those used in Meng et al.^33^ and are documented in Eqs. (11–19). Equation (11) states that each component can either include (1) or not (0) from disassembly, storage, reincarnation, reuse, recycling, or disposal. Equation (12) limits each component to only one of the remanufacturing options $x$, but at least one component needs to be disassembled and processed through ro_1_ to meet the product demand Eq. (13), but Eq. (14) ensures that the number of components disassembled is less than or equal to the total number of parts z in the assembled product *i*. Equation (16) relates to predecessors in the disassembly process. Equation (17) limits the flow of components through reuse to either reincarnation or reuse ready for sale, while Eq. (18) balances the number of components not destined for a second life with those that are recycled or disposed of. Equation (19) constrains the time functions to real numbers greater than zero.

12 $$fr_{j} ,\;fu_{j} , \;m_{j} ,\;nd_{j} , \;nsl, \;nh_{j} , \;nr_{j} , \;nu_{j} , \;nc_{j} ,\;ndp_{j} sl \in \left\{ {0,1} \right\}$$

13 $$\mathop \sum \limits_{x} ro_{jx} = 1 \quad \forall j$$

14 $$\mathop \sum \limits_{j} nd_{j} \ge ro_{j1} \ge 1$$

15 $$\mathop \sum \limits_{j} nd_{j} \le z$$

16 $$nd_{j} \ge nd_{k} \quad \forall j \in P_{k}$$

17 $$\mathop \sum \limits_{j} (fr_{j} + fu_{j} ) = 1\quad \forall j$$

18 $$nsl_{j} = \mathop \sum \limits_{j} \left( {nc_{j} + ndp_{j} } \right)$$

19 $$\lambda = TS, \;TQ_{1} , \;TQ_{2} , \;TQ_{3 } \{ \lambda \in {\mathbb{R}} |\lambda > 0\}$$

### Evaluating the environmental stewardship impact in the DMM

The potential environmental benefits, *E* result from the environmental savings made through remanufacturing a product and making it available to the customer, as opposed to one made from virgin material and processes *ES*, minus the impact to the environment from the remanufacturing function, *ERF* (Eq. 20).

20 $$E = ES - ERF$$

Referencing Fig. 2, *ERF* includes the environmental impacts of product recovery, *EPR*, disassembly, *EPD*, component storage, *ECS*, reincarnation activities, *ECR*, reuse, *ECU*, recycling, *ECC*, disposal, *ECD,* assembly, *EPA* and product finish, *EPF* (Eq. 21). *EPF* is the environmental impact of product finishing assumed to be a single value specific to the product family. Other miscellaneous environmental impacts, such as those from facility systems, will be assumed to be absorbed by others in this model.

21 $$ERF = EPR + EPD + ECS + ECR + ECU + ECC + ECD + EPA + EPF$$

Environmental impacts can be categorised as energy (J) and material consumption (kg), emissions to air and water (kg), and waste generation (kg)^36^. These can be referred to as environmental impact $e\alpha_{{\text{y}}}$, where $\alpha$ is the action (shipping, disassembly, storage, etc.) and $y \in \left\{ {1,\;2,\;3,\;4,\;5} \right\}$ reflects the impact categories. *EPR* (Eq. 22) can then be calculated from the environmental impacts of shipping es, which will likely include the energy consumption from fuel and emissions to air (Eq. 19) for the journey between the product and facility locations available from the DT.

22 $$EPR_{y} = es_{yi} l_{f}$$

*EPD* (Eq. 23) relates to the environmental impact of disassembly of component *j* per unit of time *ed* and the time it takes to disassemble *td*. Energy consumption is likely to be a key impact in both automated and semi-automated disassembly processes in remanufacturing I4.0 of the future, as electronic and/or pneumatic tooling will be prevalent and demanding of substations or compressor units. The scale of the impact will be proportional to utilisation time. Joining methods and attributes such as tightening torques can be extracted from the DT to estimate separation, tooling and fixturing energy requirements.

23 $$EPD_{1} = \mathop \sum \limits_{j} (nd_{j} ed_{y1j} td_{j} (TSsl_{j} + nsl_{j} )TQ_{1} q_{i} )$$

*ECS* (Eq. 24) results from the potential need to preserve components for storage. This can often utilise materials and generate solid waste from bagging, or if a protective coating is applied directly, emissions to water via application or energy consumption and pollutants from the wash-off process. Depending on the time in storage, multiple applications or layering of methods may be required. Material properties of components can be extracted from the ‘as designed’ DT to direct preservation methods. Therefore, if component *j* needs to be stored, *eh* is the environmental impact of holding per unit of time and *th* is the time in storage.

24 $$ECS_{y} = \mathop \sum \limits_{j} (nh_{j} eh_{yj} th_{j} )$$

The four remanufacturing option routes remain the same as in section “Evaluating the economic growth impact in the DMM”. As already presented, within the reincarnation activity, components may need to be cleaned, machined (additive or subtractive) or repaired before assembly, testing and finishing can occur. Many of these processes will come with environmental impact and the potential for all five categories being represented. Examples include powering spindles, water for coolant systems in machine tools, the heating and use of wash solutions in cleaning, the addition of new materials or replacement parts, product testing emitting emissions, wastewater and heat energy, or volatile organic compounds from paint applications. Estimations for these activities can be made by comparing the current and future state DTs.

The environmental impact per unit of time in reincarnation is *er* *ECP* (Eq. 26) is the environmental impact associated with the procurement of a part or service to replace one that will be retained for reuse, has failed, is upgradable, or is missing *ep*.

25 $$ECR_{y} = ro_{j1} = \sum\limits_{j} {nr_{j} (TQ_{2} q_{j} er_{yj} tr_{j_{yj}}) + ECP}$$

where

26 $$ECP_{y} = \sum\limits_{j} {ep_{yj} (ro_{j2} + ro_{j3} + ro_{j4} + m_{j} )}$$

Similar to the equivalent costing equations in section “Evaluating the economic growth impact in the DMM”, there could be some environmental impact generated as a result of preparing a component for reuse *eu*, and these ought to be evaluated, as reusing a product does not guarantee an environmental benefit^37^. However, these impacts are likely to be less than those associated with reincarnation, recycling and disposal, as this option is generally associated with a lower level of product change, energy expenditure and value leakage^38^. As *E* in Eq. (20) only considers the remanufactured product, *ECU* (Eq. 27) captures the environmental benefits associated with reusing the component over a new component *eru*.

27 $$ECU_{y} = ro_{j2} = \mathop \sum \limits_{j} nu_{j} (TQ_{3} eu_{yj} tu_{j} (fr_{j} + fu_{j} ) - eru_{yj} )$$

The environmental impacts of component recycling *ECC* (Eq. 28) come from the energy and materials used and wastes generated when returning a component to useable material. The total benefits associated with reusing the material over virgin material *e*rc are also considered.

28 $$ECC_{y} = ro_{j3} = \mathop \sum \limits_{j} nc_{j} (ec_{yj} - erc_{j} )$$

Similarly, the environmental impact of disposal *edp* includes emissions to water and solid waste for those components set for this route only *ndp*. As disposal is recognised as the last option in the CE loop^39^, no environmental benefits are expected. The weight of solid waste can be predicted using the material and CAD data, while the water waste estimate may need to come from a measured or inferred value, both made available in the current state DT.

29 $$ECD = ro_{j4} = \mathop \sum \limits_{j} ndp_{j} edp_{yj}$$

The environmental impacts incurred from assembly and testing, etc. are expressed in *ea*. The ‘as manufactured’ DT can support here.

30 $$EPA = \mathop \sum \limits_{j} na_{j} ea_{yj} ta_{j}$$

Constraint equations Eqs. (12–19) are applicable.

### Evaluating the social wellbeing impact in the DMM

As previously discussed, the social pillar of sustainability is the least researched to date. Therefore, the equations that drive this element of the evaluation will be based on the three distinct social groups, the employee, customer, and community^40^. The first will be based on job opportunities similar to that proposed by Meng et al.^33^, but instead of being dependent on the weight of recoverable material, it will use time. This works on the assumption that tasks requiring longer to perform than others within the scope already defined are proportional to the number of people who could be employed to complete the task. In this regard, the more people who can be employed, the better it is for society.

The second element is driven by the relationship between the customer/user and remanufacturer and made possible by the DT. With a suitable HMI, the customer can make the DT data available to EoL service providers when they no longer require the product so that remanufacturers can evaluate processing options. The remanufacturer uses these data to decide whether to recover the HiVE. This places data-driven decision-making at the forefront of remanufacturing planning, but if the remanufacturer decides not to recover the HiVE, it may become an unwanted burden to the user. This would be seen as having a negative social impact.

The final element relates to community impact and is based on the relationship between reused or remanufactured components and recycled material to those being discarded. The greater the quantity, volume or weight of material going through remanufacturing or recycling compared to disposal, the better it is for society.

Starting with the employees, of the remanufacturing activities segregated in Fig. 2, there are four that include time variables. These are disassembly, storage, reincarnation, and reuse. Assuming all man-hours *H* are valued the same, then *J* is the function that relates process time to man-hours.

31 $$H = \mathop \sum \limits_{j} J_{j} (td_{j} + ts_{j} + tr_{j} + tu_{j} )$$

With regard to the burden *B* of managing a product offered to the remanufacturing business, the impact is positive if the remanufacturing business recovers it or negative if it does not.

32 $$B = \in \left\{ { - 1,\;1} \right\}$$

Finally, the volume-based ratio of reused, remanufactured, and recycled material *V*_*r*_ to those going to disposal *V*_*d*_ is *R*.

33 $$R = \frac{{V_{r} }}{{V_{d} }}$$

A single value related to the social impact *S* is required. To ensure that each element is represented accordingly, a weighted deviation method based on Dehghanian and Mansour^41^ can be used as described in Eq. (34).

34 $$WD_{s} = \mathop \sum \limits_{n = 1}^{3} w_{n} \left( {\frac{{\left| {f_{n}^{\left( s \right)} - f_{n}^{*} } \right|}}{{f_{n}^{*} }}} \right)$$

The weighted deviation (*WD*) utilises the distance between the solution and the ideal to find the best match for the decision-maker’s requirements. If *n* represents the three elements *H*, *B* and *R*, *w*_*n*_ are the weightings applied to *n* by the business. $f_{n}^{\left( s \right)}$ and $f_{n}^{\left( * \right)}$ are the *n*th objective function values of the solution, *s*, and the ideal, *. The lower the *WD*, the closer it is to the decision-maker’s request.

This model has focused on the CE’s triple bottom line but does not include the extended ‘technological advancement’ or ‘performance management’ elements, as clarity on how these elements may be quantified is lacking in the literature. Research development, the advancement of high-tech products and conformance to guidelines, regulations and policies are all relevant and have the potential to influence the desire to remanufacture with incentives or secondary market drivers. Additionally, not considered in the model are resource allocation and availability, both of which are assumed to be finite.

### Model evaluation method

The DMM aims to optimise the disassembly sequence and provide the most cost-effective remanufacturing function for each component based on the data documented in the “Raw data” tab within Kerin et al.^42^. The raw data include a high-level list of parts and disassembly process predecessors, a component interference matrix built as per Percoco and Diella^43^ and a set of values for populating the calculation. These values assume the remanufacturing facility is based in the UK where this research was conducted.

**Figure 3:**
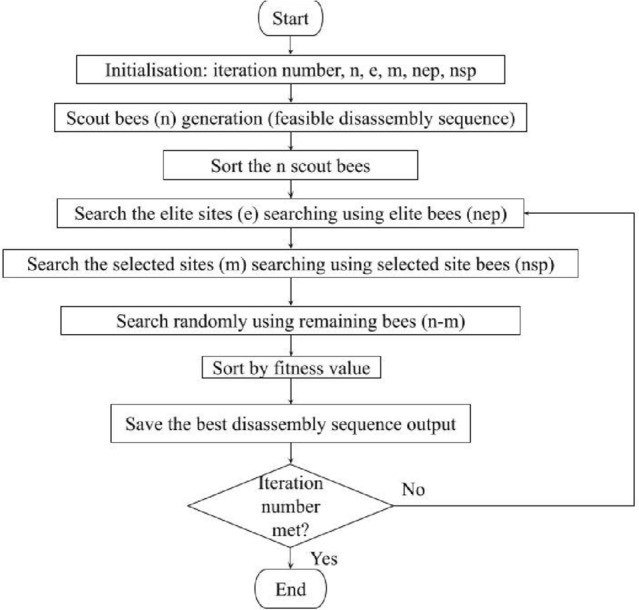
Flow chart of the Bees Algorithm used in the DT^23^.

A MATLAB version of the BA disassembly planning tool by Hartono^23^, displayed as a flow chart in Fig. 3, is utilised in the DMM to calculate the best disassembly sequence solution, the routing, and the cost associated with each targeting the minimisation of CRF.

The BA consists of five parameters that need to be set in the initialisation. In this work, the number of scout bees (*n*) = 10, the number of selected sites (*m*) = 5, the number of elite sites (*e*) = 1, the number of selected site bees (*nsp*) = 5, and the number of elite site bees (*nep*) = 10. The stopping criterion is the maximum number of iterations. The feasible disassembly sequence is generated by scout bees from the predecessor list and the component interference matrix (“Raw data” tab in Kerin et al.^42^). The *n* scout bees are sorted by their fitness values and those that are fittest are considered to have located the elite site (e) and selected sites (*m*). The *nep* bees search the elite site and its neighbourhood and the *nsp* bees forage the selected sites and their surroundings. The remaining bees (n–m) randomly explore the wider solution space. The bees are sorted by their fitness values and the best disassembly sequence plans are saved until the specified maximum iteration number is reached. The neighbourhood search strategies use swap, insert and mutation operators. The solutions consist of a disassembly sequence, disassembly recovery mode, and objective function.

## Case study

Following on from^11^ a high-value entity simulator and associated digital twin prototype (HiVE-DTP) is built using a networked Raspberry Pi (RasPi) Zero W microcontroller board connected to a DHT22 AM2302 digital temperature and humidity sensor for local ambient temperature and humidity, a BMP180 pressure sensor to measure barometric pressure and NEO-6M GPS Module with EEPROM and Built-in Active Antenna APM2.5 for location. The data collected locally are blended with publicly available whole-life performance information from^44^ to simulate an ageing, digitally connected product.

In an optimal DT solution, the virtual entity is expected to be accurate “*from a micro atomic level to the macro geometrical level*”^45^. With the technology available at this time (along with the fact that the HiVE is simulated), micro- and macro-level accuracy is unobtainable and medium fidelity is targeted. A balanced approach to the depth of twinning and the twinning rate is needed to keep the experimental system stable enough to perform (speed, processing, and data handling limitations) but also to synchronise parameters at a frequency that demonstrates the concept of the DT. Limited by the processing power of the RaspPi, the DTP is updated at least every 120 s.

The numerical demonstration utilises a large industrial engine as a case study. At approximately 2T in weight, they are of high material value with long life cycles. Many remain in service after 20 years. The information for this case study was gathered from interviews with experienced assembly and strip mechanics and process engineers, as well as from freely available technical literature and cross-referenced with data from Smith and Keoleian^36^.

**Figure 4:**
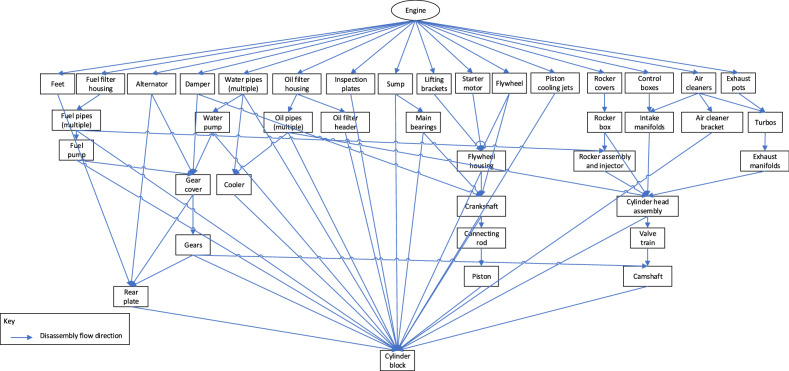
High-level disassembly precedence relationship between engine components.

The disassembly process taken by the operations team is highly dependent on the target component(s) and the state of the core. With almost 4000 components in the BoM for this case study product, the designers have worked hard to design for disassembly to facilitate servicing, so the amount of interconnectivity between the parts is limited. However, the nature of the diesel engine makes for some complicated assemblies that have been simplified significantly for this assessment. There are several different routes that can be taken in many scenarios, but the main disassembly relationship structure used in this demonstration is shown in Fig. 4.

For this case study, complete disassembly of the EoL engine core and rebuilding to form a remanufactured engine was assumed. From the relationship matrix, a list of predecessors and an interference matrix can be generated. The raw data used in the model can be found in the “Raw data” tab within Kerin et al.^42^.

### Model verification

To verify the disassembly planning tool by^23^ and its use in the DMM, the script was modified to utilise a single set of data from the HiVE-DTP with predefined component routings. Components were identified as 1–40, and their potential routings were 1 = reuse, 2 = reincarnation, 3 = recycle and 4 = dispose. The product quality was set to *q* = 0.543. The location (latitude and longitude) of the product and remanufacturing facility were provided by the DT. These were 51.5074°, 0.1278° and 52.7101°, 2.7521°, respectively. MATLAB’s Mapping Toolbox function ‘deg2km’ was used to translate these points to a singular distance value in km. In parallel, a manual assessment (see “Supporting info and Manual Ass.” tab in Kerin et al.^42^) of the same data was performed. The results were compared and found to be identical. The CRF from both the computed model and manual assessment was £29,058.84. With a newly manufactured product sales price of approximately £38,000, a remanufactured goods sales price of 20–40% below^46^ would result in a target selling price of £26,600 (70% of new).

The above validation exercise demonstrated (1) that the model was functioning correctly and (2) its proximity to the target selling price suggested it had the potential to generate profitable solutions with the raw data available. Two runs of the DMM are displayed in Table 1, including the sequence of disassembly, routing of components and the associated costs. Both disassembly sequences were assessed and declared viable.

**Table 1:** DMM algorithm output and validation.

Result using fix route (mode U = 1, R = 2, C = 3, D = 4)																																								
Run 1																																								
Sequence	27	16	38	25	5	14	36	13	34	28	37	9	11	35	39	4	32	30	33	23	24	19	3	29	15	40	21	22	18	31	17	20	2	26	10	7	6	12	8	1
Mode	4	1	3	3	4	2	4	3	3	2	3	1	2	4	1	1	1	1	3	3	3	1	4	3	3	1	3	3	3	1	3	3	2	1	2	2	3	2	3	2
BestCost (£)	29,058.44																																							
Run 2																																								
Sequence	5	38	30	13	25	24	27	28	23	32	9	17	26	16	15	40	39	4	14	36	35	33	29	22	21	34	37	19	20	3	18	11	31	10	2	7	6	8	12	1
Mode	4	3	1	3	3	3	4	2	3	1	1	3	1	1	3	1	1	1	2	4	4	3	3	3	3	3	3	1	3	4	3	2	1	2	2	2	3	3	2	2
BestCost (£)	29,058.44																																							

### Model optimisation

In a fully automated disassembly cell, the sequence, as long as it was viable, would matter less as the system memory would manage the process and have awareness of all components and product build level. However, the process data used in this case study are based on human disassembly. While the sequences generated and presented in Table 1 were viable, they did not offer a logical structure for human-managed disassembly. Following the sequence suggested by the algorithm would demand the operator to change the direction and logical flow of the work content. To weigh the disassembly process to drive a more human-friendly solution, a disassembly matrix was generated that applied a penalty to a change in disassembly direction of + 1 min for 90° and + 2 min for 180°. The disassembly time matrix can be found in the “Disassembly time matrix” tab in Kerin et al.^42^.

In addition, the model was designed to allow the remanufacturing routings to be managed in two ways. First, a ‘fixed’ mode that enabled the routings to be predefined would suit already established or flexible processes. This mode was used to validate the results with the manual process. Second, a ‘self-assigned’ mode works to identify the best routing available from the potential options, as defined in the raw data, suitable for flexible disassembly and management processes. The results in section “Results” utilise the ‘self-assigned’ mode.

## Results

All results are documented in the ‘Results’ tab in Kerin et al.^42^. A sample of the DMM output at *q* = 1 is presented in Tables 2 and 3.

**Table 2:** BA-generated disassembly sequence results at q = 1.

Quality (q)	Distance (km)	Disassembly sequence (by part number)																																						
1	0	13	32	17	35	9	15	39	24	38	25	16	21	14	30	11	26	5	10	4	23	22	40	36	7	34	27	3	33	28	20	2	37	29	8	6	19	18	12	31
1	100	39	38	14	13	9	5	32	25	17	27	33	35	4	24	26	30	16	11	15	23	28	22	10	20	40	36	7	34	3	21	29	8	37	2	19	18	6	12	31
1	200	39	13	27	33	9	15	35	38	40	16	22	11	36	34	25	24	14	30	5	21	17	37	4	20	28	23	3	32	29	2	6	26	10	19	7	18	12	31	8
1	300	39	24	35	16	17	38	5	27	14	28	32	30	4	9	13	23	11	15	33	25	40	22	20	26	29	10	36	7	34	8	37	19	21	3	2	18	6	31	12
1	400	39	35	14	27	16	36	38	34	33	37	13	25	5	17	4	9	15	24	26	21	30	32	22	20	11	10	3	40	28	23	2	6	29	19	7	8	18	12	31
1	500	39	36	24	5	13	14	34	17	37	38	9	35	16	27	33	32	4	28	11	15	3	22	40	21	20	25	26	10	7	8	2	30	23	19	18	29	31	6	12
1	600	39	35	5	24	25	13	4	27	9	15	38	16	32	17	26	22	30	21	14	36	11	20	28	33	34	40	37	29	10	7	8	3	23	2	6	19	18	31	12
1	700	39	14	24	17	32	5	13	25	26	4	35	16	9	38	30	11	15	22	40	10	36	7	27	21	28	8	34	23	3	37	33	29	20	2	19	6	18	31	12
1	800	13	39	9	36	38	17	32	34	16	27	37	11	15	24	40	14	35	5	30	22	33	28	4	25	21	20	3	23	19	29	26	18	2	31	10	6	12	7	8
1	900	13	5	25	4	24	9	30	17	39	14	23	16	26	11	10	15	36	7	34	35	3	27	22	8	37	28	19	33	40	29	32	20	38	21	2	18	31	6	12
1	1000	13	39	5	32	17	38	9	27	24	4	35	25	11	15	16	22	40	26	20	33	30	28	21	29	14	23	10	36	7	8	34	3	37	2	6	19	18	31	12
1	1100	39	13	30	35	17	23	16	25	26	14	9	15	38	22	40	24	20	5	36	34	37	11	4	10	19	7	3	32	27	28	18	31	33	29	8	21	2	6	12
1	1200	39	24	27	16	35	9	11	15	36	40	25	14	30	5	4	21	32	22	33	28	17	20	13	26	29	34	38	37	23	10	7	19	3	2	8	6	18	12	31
1	1300	13	36	39	30	14	24	27	34	35	9	5	11	15	37	16	17	33	22	32	38	4	20	28	23	3	21	19	29	2	40	25	6	18	31	12	26	10	7	8
1	1400	13	25	39	5	24	36	14	30	35	38	16	27	23	9	34	28	11	15	17	40	37	21	19	33	4	32	3	26	10	22	18	7	20	29	2	31	6	8	12
1	1500	39	14	27	17	33	36	24	30	23	9	15	11	34	37	35	21	16	5	32	13	38	28	4	25	22	40	26	3	10	20	29	7	8	19	18	2	6	12	31
1	1600	39	27	14	16	9	15	17	24	22	38	36	34	5	32	11	30	23	35	13	37	19	18	21	28	33	29	4	40	31	25	20	26	10	3	7	8	2	6	12
1	1700	13	27	9	15	33	17	11	24	25	5	4	38	26	14	35	21	16	10	36	7	40	22	34	8	3	37	20	30	23	28	39	2	29	19	6	32	18	31	12
1	1800	13	25	17	39	5	27	35	33	14	30	23	24	9	11	15	28	4	16	40	22	20	29	38	26	10	36	7	34	37	32	8	3	19	18	31	21	2	6	12
1	1900	39	35	13	17	24	36	27	30	38	32	16	34	33	25	14	37	9	15	28	26	21	23	11	29	22	19	20	5	18	10	4	7	31	3	8	2	6	40	12
1	2000	39	36	14	27	33	24	16	13	9	35	30	23	25	32	28	34	29	11	15	17	40	22	21	5	37	20	4	3	2	19	38	18	6	12	26	31	10	7	8
1	2100	13	24	30	23	14	17	36	32	27	33	28	35	39	9	15	11	34	38	16	37	19	18	25	40	5	21	22	4	29	26	31	20	3	2	6	10	12	7	8
1	2200	39	36	5	27	33	4	35	9	15	11	34	32	17	13	16	14	22	38	25	3	21	30	26	28	23	20	29	37	24	2	40	10	19	6	18	31	7	12	8
1	2300	39	9	25	27	28	13	11	15	32	30	24	14	16	40	22	23	35	5	4	21	38	33	36	29	34	17	26	20	10	3	37	19	18	31	2	7	6	12	8
1	2400	39	16	24	32	30	36	23	9	27	11	15	17	33	5	25	38	34	37	35	21	14	19	40	22	13	28	29	20	26	10	18	31	7	8	4	3	2	6	12
1	2500	13	35	9	17	39	32	14	11	15	30	25	38	26	5	40	16	22	10	36	7	23	4	27	24	20	33	21	34	3	2	8	6	28	37	19	29	18	12	31
1	2600	39	35	9	38	11	15	24	17	27	33	36	28	5	30	23	16	4	29	21	34	14	37	40	32	3	19	22	20	2	6	25	18	31	12	13	26	10	7	8
1	2700	39	27	38	5	24	9	15	16	13	32	11	25	14	28	33	40	4	22	17	30	23	29	35	20	26	21	10	36	7	8	34	3	37	2	6	19	18	31	12
1	2800	13	36	39	30	38	34	37	23	25	14	17	35	26	24	5	9	27	32	33	28	4	29	3	11	15	19	40	16	10	22	7	18	20	31	21	8	2	6	12
1	2900	39	13	24	17	14	30	27	23	5	16	25	33	35	38	9	28	11	26	4	10	15	36	7	29	40	8	22	21	32	20	34	3	2	6	37	19	18	12	31
1	3000	39	5	16	25	27	33	32	36	35	28	34	38	37	9	15	22	24	40	17	21	14	4	30	3	20	13	26	2	6	29	23	19	18	12	11	10	31	7	8
1	3100	13	25	39	5	9	15	16	38	32	11	30	17	27	24	26	28	33	35	14	40	23	10	36	7	29	22	4	20	8	21	34	3	37	2	6	19	18	31	12
1	3200	13	27	30	17	32	39	14	28	33	9	29	11	15	24	38	25	23	26	35	21	40	16	5	10	36	7	8	22	4	20	34	37	3	2	6	19	18	12	31
1	3300	13	16	25	32	35	5	38	36	39	24	34	9	17	26	27	28	11	15	37	22	30	20	40	21	23	14	33	19	18	29	10	7	4	31	8	3	2	6	12
1	3400	39	35	9	15	16	17	24	22	27	40	13	32	30	38	5	25	14	4	20	11	26	28	33	10	29	36	7	8	21	34	23	3	37	19	18	31	2	6	12
1	3500	13	17	25	9	27	16	24	28	35	14	26	5	33	32	11	10	15	38	40	4	21	22	20	39	36	7	30	8	29	34	3	37	23	19	2	18	6	31	12
1	3600	39	5	14	32	4	36	13	9	15	30	25	17	35	26	38	16	34	27	28	3	40	22	20	24	37	23	19	33	21	29	11	10	7	8	18	31	2	6	12
1	3700	13	9	39	38	30	32	27	28	24	11	15	17	25	5	16	23	26	14	4	10	35	22	21	20	40	33	36	7	34	8	29	37	19	3	18	31	2	6	12
1	3800	39	25	17	16	24	27	5	13	38	32	28	4	9	14	11	15	26	30	10	22	40	36	7	33	8	29	20	35	34	21	37	23	3	2	6	19	18	31	12
1	3900	39	5	24	16	32	30	4	27	14	25	9	17	11	15	13	36	28	35	26	38	22	23	33	29	34	20	21	3	37	10	7	8	40	2	6	19	18	31	12
1	4000	39	9	27	14	30	38	35	32	13	16	24	11	17	25	23	26	10	15	36	7	33	21	28	34	37	8	40	29	19	18	31	5	4	3	22	20	2	6	12
1	4100	13	17	9	15	38	36	27	32	39	5	40	35	16	28	25	22	30	23	14	26	11	21	20	4	10	7	24	33	8	29	34	3	37	2	6	19	18	31	12
1	4200	39	16	27	9	15	5	40	25	32	13	22	38	4	35	14	36	21	11	34	28	33	30	24	3	23	29	17	26	10	7	8	37	19	18	31	20	2	6	12
1	4300	13	9	16	27	28	38	30	36	17	25	26	35	32	11	15	24	40	33	5	4	29	34	39	14	23	37	10	3	22	20	21	19	7	18	31	2	8	6	12
1	4400	13	5	16	35	17	9	27	38	11	15	14	32	33	4	39	21	25	26	10	22	28	40	24	30	20	29	23	36	7	34	3	2	8	37	6	19	18	12	31
1	4500	13	25	32	27	16	38	28	5	24	39	36	17	26	9	15	22	14	4	40	33	30	11	35	34	29	20	37	3	10	21	2	7	6	8	23	19	18	12	31
1	4600	39	17	27	36	9	15	25	38	28	34	40	30	13	23	37	14	16	24	35	21	5	26	4	22	20	32	19	3	33	18	29	11	10	7	2	31	6	8	12
1	4700	39	36	17	34	9	16	32	35	27	28	14	24	25	5	11	15	4	30	22	40	13	38	23	37	3	19	18	26	10	31	21	20	7	8	33	2	29	6	12
1	4800	13	17	25	9	15	35	26	16	27	30	22	23	11	14	5	39	4	33	28	10	32	40	36	7	24	38	20	34	21	3	29	2	37	19	18	8	31	6	12
1	4900	13	35	25	9	15	36	14	27	34	11	17	16	38	33	40	24	30	32	37	22	23	21	39	28	29	26	5	10	7	8	4	20	3	19	18	31	2	6	12
1	5000	39	27	17	28	32	24	13	5	38	25	35	9	15	11	33	21	26	30	29	23	14	10	16	36	7	4	22	34	40	37	20	19	18	31	3	2	8	6	12

**Table 3:** BA-generated routing and costs related to the sequences in Table 2.

Quality (q)	Distance (km)	Remanufacturing method (1 = Reuse, 2 = Reincarnate, 3 = Recycle, 4 = Disposal)																																								Cost (£)
1	0	3	1	3	4	1	3	1	3	3	3	1	3	2	1	2	1	4	2	1	3	3	1	4	2	3	4	4	3	2	3	2	3	3	3	3	1	3	2	1	2	23,921
1	100	1	3	2	3	1	4	1	3	3	4	3	4	1	3	1	1	1	2	3	3	2	3	2	3	1	4	2	3	4	3	3	3	3	2	1	3	3	2	1	2	23,937
1	200	1	3	4	3	1	3	4	3	1	1	3	2	4	3	3	3	2	1	4	3	3	3	1	3	2	3	4	1	3	2	3	1	2	1	2	3	2	1	3	2	23,957
1	300	1	3	4	1	3	3	4	4	2	2	1	1	1	1	3	3	2	3	3	3	1	3	3	1	3	2	4	2	3	3	3	1	3	4	2	3	3	1	2	2	23,969
1	400	1	4	2	4	1	4	3	3	3	3	3	3	4	3	1	1	3	3	1	3	1	1	3	3	2	2	4	1	2	3	2	3	3	1	2	3	3	2	1	2	23,989
1	500	1	4	3	4	3	2	3	3	3	3	1	4	1	4	3	1	1	2	2	3	4	3	1	3	3	3	1	2	2	3	2	1	3	1	3	3	1	3	2	2	24,005
1	600	1	4	4	3	3	3	1	4	1	3	3	1	1	3	1	3	1	3	2	4	2	3	2	3	3	1	3	3	2	2	3	4	3	2	3	1	3	1	2	2	24,021
1	700	1	2	3	3	1	4	3	3	1	1	4	1	1	3	1	2	3	3	1	2	4	2	4	3	2	3	3	3	4	3	3	3	3	2	1	3	3	1	2	2	24,032
1	800	3	1	1	4	3	3	1	3	1	4	3	2	3	3	1	2	4	4	1	3	3	2	1	3	3	3	4	3	1	3	1	3	2	1	2	3	2	2	3	2	24,053
1	900	3	4	3	1	3	1	1	3	1	2	3	1	1	2	2	3	4	2	3	4	4	4	3	3	3	2	1	3	1	3	1	3	3	3	2	3	1	3	2	2	24,064
1	1000	3	1	4	1	3	3	1	4	3	1	4	3	2	3	1	3	1	1	3	3	1	2	3	3	2	3	2	4	2	3	3	4	3	2	3	1	3	1	2	2	24,080
1	1100	1	3	1	4	3	3	1	3	1	2	1	3	3	3	1	3	3	4	4	3	3	2	1	2	1	2	4	1	4	2	3	1	3	3	3	3	2	3	2	2	24,100
1	1200	1	3	4	1	4	1	2	3	4	1	3	2	1	4	1	3	1	3	3	2	3	3	3	1	3	3	3	3	3	2	2	1	4	2	3	3	3	2	1	2	24,116
1	1300	3	4	1	1	2	3	4	3	4	1	4	2	3	3	1	3	3	3	1	3	1	3	2	3	4	3	1	3	2	1	3	3	3	1	2	1	2	2	3	2	24,132
1	1400	3	3	1	4	3	4	2	1	4	3	1	4	3	1	3	2	2	3	3	1	3	3	1	3	1	1	4	1	2	3	3	2	3	3	2	1	3	3	2	2	24,148
1	1500	1	2	4	3	3	4	3	1	3	1	3	2	3	3	4	3	1	4	1	3	3	2	1	3	3	1	1	4	2	3	3	2	3	1	3	2	3	2	1	2	24,164
1	1600	1	4	2	1	1	3	3	3	3	3	4	3	4	1	2	1	3	4	3	3	1	3	3	2	3	3	1	1	1	3	3	1	2	4	2	3	2	3	2	2	24,180
1	1700	3	4	1	3	3	3	2	3	3	4	1	3	1	2	4	3	1	2	4	2	1	3	3	3	4	3	3	1	3	2	1	2	3	1	3	1	3	1	2	2	24,191
1	1800	3	3	3	1	4	4	4	3	2	1	3	3	1	2	3	2	1	1	1	3	3	3	3	1	2	4	2	3	3	1	3	4	1	3	1	3	2	3	2	2	24,207
1	1900	1	4	3	3	3	4	4	1	3	1	1	3	3	3	2	3	1	3	2	1	3	3	2	3	3	1	3	4	3	2	1	2	1	4	3	2	3	1	2	2	24,227
1	2000	1	4	2	4	3	3	1	3	1	4	1	3	3	1	2	3	3	2	3	3	1	3	3	4	3	3	1	4	2	1	3	3	3	2	1	1	2	2	3	2	24,243
1	2100	3	3	1	3	2	3	4	1	4	3	2	4	1	1	3	2	3	3	1	3	1	3	3	1	4	3	3	1	3	1	1	3	4	2	3	2	2	2	3	2	24,259
1	2200	1	4	4	4	3	1	4	1	3	2	3	1	3	3	1	2	3	3	3	4	3	1	1	2	3	3	3	3	3	2	1	2	1	3	3	1	2	2	3	2	24,275
1	2300	1	1	3	4	2	3	2	3	1	1	3	2	1	1	3	3	4	4	1	3	3	3	4	3	3	3	1	3	2	4	3	1	3	1	2	2	3	2	3	2	24,291
1	2400	1	1	3	1	1	4	3	1	4	2	3	3	3	4	3	3	3	3	4	3	2	1	1	3	3	2	3	3	1	2	3	1	2	3	1	4	2	3	2	2	24,307
1	2500	3	4	1	3	1	1	2	2	3	1	3	3	1	4	1	1	3	2	4	2	3	1	4	3	3	3	3	3	4	2	3	3	2	3	1	3	3	2	1	2	24,318
1	2600	1	4	1	3	2	3	3	3	4	3	4	2	4	1	3	1	1	3	3	3	2	3	1	1	4	1	3	3	2	3	3	3	1	2	3	1	2	2	3	2	24,339
1	2700	1	4	3	4	3	1	3	1	3	1	2	3	2	2	3	1	1	3	3	1	3	3	4	3	1	3	2	4	2	3	3	4	3	2	3	1	3	1	2	2	24,350
1	2800	3	4	1	1	3	3	3	3	3	2	3	4	1	3	4	1	4	1	3	2	1	3	4	2	3	1	1	1	2	3	2	3	3	1	3	3	2	3	2	2	24,371
1	2900	1	3	3	3	2	1	4	3	4	1	3	3	4	3	1	2	2	1	1	2	3	4	2	3	1	3	3	3	1	3	3	4	2	3	3	1	3	2	1	2	24,382
1	3000	1	4	1	3	4	3	1	4	4	2	3	3	3	1	3	3	3	1	3	3	2	1	1	4	3	3	1	2	3	3	3	1	3	2	2	2	1	2	3	2	24,402
1	3100	3	3	1	4	1	3	1	3	1	2	1	3	4	3	1	2	3	4	2	1	3	2	4	2	3	3	1	3	3	3	3	4	3	2	3	1	3	1	2	2	24,414
1	3200	3	4	1	3	1	1	2	2	3	1	3	2	3	3	3	3	3	1	4	3	1	1	4	2	4	2	3	3	1	3	3	3	4	2	3	1	3	2	1	2	24,430
1	3300	3	1	3	1	4	4	3	4	1	3	3	1	3	1	4	2	2	3	3	3	1	3	1	3	3	2	3	1	3	3	2	2	1	1	3	4	2	3	2	2	24,450
1	3400	1	4	1	3	1	3	3	3	4	1	3	1	1	3	4	3	2	1	3	2	1	2	3	2	3	4	2	3	3	3	3	4	3	1	3	1	2	3	2	2	24,462
1	3500	3	3	3	1	4	1	3	2	4	2	1	4	3	1	2	2	3	3	1	1	3	3	3	1	4	2	1	3	3	3	4	3	3	1	2	3	3	1	2	2	24,477
1	3600	1	4	2	1	1	4	3	1	3	1	3	3	4	1	3	1	3	4	2	4	1	3	3	3	3	3	1	3	3	3	2	2	2	3	3	1	2	3	2	2	24,498
1	3700	3	1	1	3	1	1	4	2	3	2	3	3	3	4	1	3	1	2	1	2	4	3	3	3	1	3	4	2	3	3	3	3	1	4	3	1	2	3	2	2	24,509
1	3800	1	3	3	1	3	4	4	3	3	1	2	1	1	2	2	3	1	1	2	3	1	4	2	3	3	3	3	4	3	3	3	3	4	2	3	1	3	1	2	2	24,525
1	3900	1	4	3	1	1	1	1	4	2	3	1	3	2	3	3	4	2	4	1	3	3	3	3	3	3	3	3	4	3	2	2	3	1	2	3	1	3	1	2	2	24,545
1	4000	1	1	4	2	1	3	4	1	3	1	3	2	3	3	3	1	2	3	4	2	3	3	2	3	3	3	1	3	1	3	1	4	1	4	3	3	2	3	2	2	24,557
1	4100	3	3	1	3	3	4	4	1	1	4	1	4	1	2	3	3	1	3	2	1	2	3	3	1	2	2	3	3	3	3	3	4	3	2	3	1	3	1	2	2	24,577
1	4200	1	1	4	1	3	4	1	3	1	3	3	3	1	4	2	4	3	2	3	2	3	1	3	4	3	3	3	1	2	2	3	3	1	3	1	3	2	3	2	2	24,593
1	4300	3	1	1	4	2	3	1	4	3	3	1	4	1	2	3	3	1	3	4	1	3	3	1	2	3	3	2	4	3	3	3	1	2	3	1	2	3	3	2	2	24,609
1	4400	3	4	1	4	3	1	4	3	2	3	2	1	3	1	1	3	3	1	2	3	2	1	3	1	3	3	3	4	2	3	4	2	3	3	3	1	3	2	1	2	24,621
1	4500	3	3	1	4	1	3	2	4	3	1	4	3	1	1	3	3	2	1	1	3	1	2	4	3	3	3	3	4	2	3	2	2	3	3	3	1	3	2	1	2	24,641
1	4600	1	3	4	4	1	3	3	3	2	3	1	1	3	3	3	2	1	3	4	3	4	1	1	3	3	1	1	4	3	3	3	2	2	2	2	1	3	3	2	2	24,657
1	4700	1	4	3	3	1	1	1	4	4	2	2	3	3	4	2	3	1	1	3	1	3	3	3	3	4	1	3	1	2	1	3	3	2	3	3	2	3	3	2	2	24,673
1	4800	3	3	3	1	3	4	1	1	4	1	3	3	2	2	4	1	1	3	2	2	1	1	4	2	3	3	3	3	3	4	3	2	3	1	3	3	1	3	2	2	24,684
1	4900	3	4	3	1	3	4	2	4	3	2	3	1	3	3	1	3	1	1	3	3	3	3	1	2	3	1	4	2	2	3	1	3	4	1	3	1	2	3	2	2	24,704
1	5000	1	4	3	2	1	3	3	4	3	3	4	1	3	2	3	3	1	1	3	3	2	2	1	4	2	1	3	3	1	3	3	1	3	1	4	2	3	3	2	2	24,716

As seen in Table 2, the BA works to identify the disassembly sequence that provides the cheapest remanufacturing function cost for the inputs provided. Similarly, it looks to optimise the routing of the components to minimise expenditure (Table 3).

**Figure 5:**
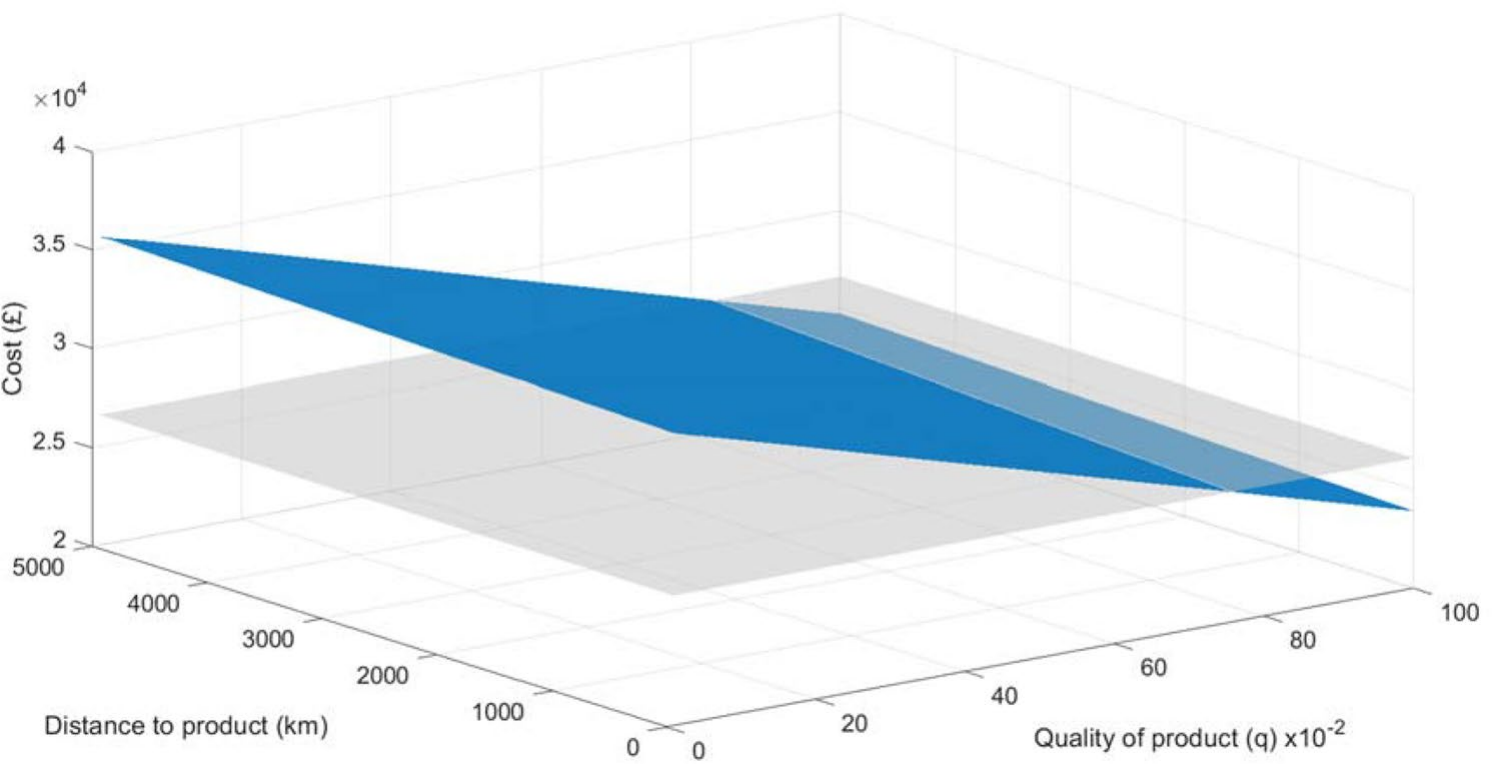
Results of DMM.

The DMM was run with quality varying by 0.1 units from 0 to 1.0 and distance by 100 km from 0 to 5000 km to evaluate the relationships that would determine whether the product should be recovered for remanufacturing. Working with a breakeven of £26,600, Fig. 5 displays the effects of changing quality and distance between the product and remanufacturing facility on the cost to complete the remanufacturing function, CRF.

Using this set of raw data, there appears to be little opportunity to recover and make a profit out of the remanufacturing of this product, identified as the area below the grey breakeven plane. At $q \le 0.7$, the DT would suggest that the product was not worth recovering and that the user should look to find an alternative EoL solution for it. At $q > 0.8$, there could well be an opportunity to generate a profit and fulfil a customer order. At $q = 0.8$, the influence of distance is seen with those products closer to the facility profitable, in contrast to those located further away.

As the raw data were generated from a multitude of sources, there may be a case for caution over the exact resultant values; however, what has been demonstrated is that the model can be run in the DT to provide a numerical assessment of the potential profits obtainable from a product in MoL using the DT as the conduit that will enable a data-driven decision by the remanufacturers as to whether or not a product should be recovered and how best it may be processed.

## Discussion

The output of the DMM provides evidence that a DT can provide valuable information for remanufacturing businesses. It offers an automated assessment of a product’s potential to generate revenue assuming the product is in demand. For a fully flexible, highly automated disassembly and rebuilding process, the BA provides a solution that can be used to drive the remanufacturing process plan and enable accurate forecasting to feed forwards replacement part purchasing and reusable components or remanufactured product sales.

The implementation of the directional change penalty matrix to support a more human manageable process to match the case study inputs did offer a slightly more logical disassembly process when discussed with skilled operators, but ultimately, this element needs more consideration and could be an interesting progression of this work. Should this model be applied to other applications, more emphasis needs to be placed on this element of the remanufacturing process and the inputs available from the DT process. In this work, only the inputs from the product DT were truly considered.

The environmental and social elements of the model have not been tested as part of this research, as their similarity to the economic element meant doing so would add little value. The BA has already been proven to solve multiobjective optimisation problems^47^ and could be used to resolve the complete model. Raw data for the environmental and social elements could also be difficult to capture without the comprehensive engagement of a remanufacturing business.

## Conclusion

Returning to the questions posed at the beginning of this research, the key variables that need to be incorporated into the DMM relate to product quality, location, and remanufacturing costs. If the remanufacturing business is to be managed to CE principles, the outputs of the DT should feed into a triple-bottom-line assessment. The decision whether to recover an EoL product would then be achieved through evaluating the financial, environmental, and social costs of the recovery and disassembly, component storage, component/product reincarnation activities, recycling, and disposal, along with product assembly, testing, and finishing.

This article has documented the realisation of a digital twin (DT) sub-element, the decision-making module (DMM) of a smart remanufacturing system. The inputs and outputs to the DMM along with the assumptions and relationships have been defined. From this, a model structure is proposed that utilises three of the nine CE-I4.0 methodologies: reuse, remanufacture and recycle. The fourth route used is disposal.

Based on the three pillars of the CE, the article offers a numerical assessment of the product and EoL options. To test the model, the economic pillar was used, and a case study was conducted. Targeting the lowest incurred costs, the optimal disassembly sequence and component routing were found using the Bess Algorithm. This enables near real-time data-driven decisions to be made for a product almost at the end of its service life that is still in use and emitting sensor readings. Combined with the RUL calculations, this information can give remanufacturing businesses foresight into the current quality of the product, degradation characteristics and remanufacturing potential.

There are a number of further research opportunities including expansion of the directional change penalty matrix to include the impact due to the change in tooling, the testing of the social and environmental elements of the model using real data, trialling other high-value entities, and evaluating the impact to the DT inputs available when employing the model on a different remanufacturing process. Finally, the subject of virtual-physical synchronisation, depth of data and frequency of sampling and feedback required by a DT to be sufficiently timely for remanufacturers needs more research.

## Data Availability

All data generated or analysed during this study are included in the UBIRA eData repository, at https://doi.org/10.25500/eData.bham.00000855.

## Abbreviations

α: An action in the remanufacturing function that causes an environmental impact (shipping, disassembly, storage etc.)
λ: Time functions
B: Management of unwanted asset (burden)
BA: Bees Algorithm
BoL: Beginning of Life
BoP: Bill of Process
ca: Cost of assembling product per unit of time
cc: Cost of recycling component
CCA: Cost of component assembly
CCC: Cost of component recycling
CCD: Cost of component disposal
CCR: Cost of component reincarnation
CCS: Cost of component storage
CCU: Cost of component reuse
cd: Cost of disassembly per unit of time
cdp: Cost of disposal
CE: Circular Economy
ch: Cost of holding component per unit of time
cp: Cost of procuring component
CPD: Cost of product disassembly
CPF: Cost of product finishing
cph: Cost of preserving component for storage
CPR: Cost of product recovery
cr: Cost of reincarnating component per unit of time
CRF: Cost of remanufacturing function
cru: Component revenue from reuse
cs: Cost of shipping per km
cu: Cost of preparing component for reusing per unit of time
DMM: Decision-Making Module
DT: Digital Twins
DTA: Digital Twin Aggerate
DTE: Digital Twin Environment
DTI: Digital Twin Instance
DTP: Digital Twin Prototype
E: Environmental benefits
eBoM: Engineering Bill of Materials
ECA: Environmental impacts of component assembly
ECC: Environmental impacts of component recycling
ECD: Environmental impacts of component disposal
ECP: Environmental impacts of component procurement
ECR: Environmental impacts of component reincarnation activities
ECS: Environmental impacts of component storage
ECU: Environmental impacts of reuse
ed: Environmental impact of disassembly of component per unit of time
edp: Environmental impact of disposal
eh: Environmental impact of holding per unit of time
EoL: End of Life
ep: Environmental impact associated with the procurement of a part
EPD: Environmental impacts of product disassembly
EPR: Environmental impacts of product recovery
er: Environmental impact per unit of time in reincarnation
ERF: Environmental impact from remanufacturing function
erc: Environmental benefits associated with reusing the material over virgin material
eru: Environmental benefits associated with reusing the component over a new component
es: Environmental impacts of shipping
ES: Environmental savings
eu: Environmental impact as a result of preparing a component for reuse
f: Remanufacturing facility
fr: Routes through to reincarnation from reuse
fu: Routes through to available for sale from reuse
GPS: Global Positioning System
H: Number of man-hours
HiVE: High-value entity
HMI: Human–Machine Interface
i: Product
I4.0: Industry 4.0
IoT: Internet of Things
j, k..z: Components
J: Function representing the link between process time and man-hours
l: Distance in km
m: Missing component
MoL: Middle of Life
mrc: Material revenue from recycling
NA: Nature-inspired Algorithms
nc: Need to recycle
nd: Need to disassemble component 1 or 0
ndp: Need for disposal
nh: Need to hold component
nr: Need to reincarnate component
nsl: Component destined for recycling or disposal
Nu: Need to reuse
OS: Optimal solution
qi, qj: Product quality, component quality
qmin: Min quality of component for use in remanufactured product
RasPi: Raspberry Pi
rBoM: Remanufacturing Bill of Materials
rBoP: Remanufacturing Bill of Process
ro: Routes through the remanufacturing function
sl: Component destined for reincarnation or reuse
SP: Sales price of the remanufactured product
SRDM: Smart Recovery Decision-Making
ta: Time to assemble product
td: Time it takes to disassemble
th: Time component in storage
TQ1: Function representing impact of q on disassembly time
TQ2: Function representing impact of q on reincarnation time
TQ3: Function representing impact of q on reuse time
tr: Time to reincarnate component
TS: Function representing impact of second-life destination on disassembly time
tu: Time to prepare component for reuse
WD: Weighted deviation
x: Represents reincarnation, reuse, recycling, or disposal (1, 2, 3 or 4)
y: Impact categories (energy, material consumption, emissions to air and water, waste generation)
